# T cell immunity ameliorates COVID-19 disease severity and provides post-exposure prophylaxis after peptide-vaccination, in Syrian hamsters

**DOI:** 10.3389/fimmu.2023.1111629

**Published:** 2023-01-24

**Authors:** Eszter Somogyi, Mariann Kremlitzka, Zsolt Csiszovszki, Levente Molnár, Orsolya Lőrincz, József Tóth, Leon de Waal, Sofie Pattijn, Wencke Reineking, Andreas Beineke, Enikő R. Tőke

**Affiliations:** ^1^ Treos Bio Ltd, London, United Kingdom; ^2^ Treos Bio Zrt, Veszprém, Hungary; ^3^ PepTC Vaccines Ltd, London, United Kingdom; ^4^ Viroclinics Biosciences B.V., Viroclinics Xplore, Schaijk, Netherlands; ^5^ ImmunXperts Société Anonyme, Q2 Solutions Company, Gosselies, Belgium; ^6^ Department of Pathology, University of Veterinary Medicine Hannover, Hannover, Germany

**Keywords:** T cells, post-exposure prophylaxis, therapeutic, vaccine, SARS-CoV-2, adaptive immunity

## Abstract

**Background:**

The emergence of novel SARS-CoV-2 variants that resist neutralizing antibodies drew the attention to cellular immunity and calls for the development of alternative vaccination strategies to combat the pandemic. Here, we have assessed the kinetics of T cell responses and protective efficacy against severe COVID-19 in pre- and post-exposure settings, elicited by PolyPEPI-SCoV-2, a peptide based T cell vaccine.

**Methods:**

75 Syrian hamsters were immunized subcutaneously with PolyPEPI-SCoV-2 on D0 and D14. On D42, hamsters were intranasally challenged with 10^2^ TCID_50_ of the virus. To analyze immunogenicity by IFN-γ ELISPOT and antibody secretion, lymphoid tissues were collected both before (D0, D14, D28, D42) and after challenge (D44, D46, D49). To measure vaccine efficacy, lung tissue, throat swabs and nasal turbinate samples were assessed for viral load and histopathological changes. Further, body weight was monitored on D0, D28, D42 and every day after challenge.

**Results:**

The vaccine induced robust activation of T cells against all SARS-CoV-2 structural proteins that were rapidly boosted after virus challenge compared to control animals (~4-fold, p<0.05). A single dose of PolyPEPI-SCoV-2 administered one day after challenge also resulted in elevated T cell response (p<0.01). The vaccination did not induce virus-specific antibodies and viral load reduction. Still, peptide vaccination significantly reduced body weight loss (p<0.001), relative lung weight (p<0.05) and lung lesions (p<0.05), in both settings.

**Conclusion:**

Our study provides first proof of concept data on the contribution of T cell immunity on disease course and provide rationale for the use of T cell-based peptide vaccines against both novel SARS-CoV-2 variants and supports post-exposure prophylaxis as alternative vaccination strategy against COVID-19.

## 1 Introduction

Since the first report of severe acute respiratory syndrome coronavirus-2 (SARS-CoV-2) infection in December 2019, the virus spread with an enormous rate causing over 637 million cases and more than 6.6 million death worldwide until November 2022 according to the World Health Organization (WHO) coronavirus dashboard (1). Repeated SARS-CoV-2 vaccination has proved to be effective in prevention of COVID-19, however recent reports show that antibody-mediated immunity is waning – especially against novel variants of concern (VOC) (2). Although vaccines against currently circulating Omicron subvariants (BA.1, BA.4 and BA.5) have been authorized or recommended for authorization (3, 4), the currently available COVID-19 vaccines encode mainly the S protein of the virus and hence, are sensitive for the continuous mutation of SARS-CoV-2. Therefore, new vaccines providing long-term protection against recently emerging variants would be still required.

Alternative solution would be to leverage disease-modifying strategies in order to prevent new severe COVID-19 cases and control the pandemic. Currently, following SARS-CoV-2 exposure, mainly isolation of infected individuals is applied and therapeutic treatments show limited benefit (5). One of the potential solutions would be the post-exposure prophylaxis (PEP) used to attenuate the infection-related symptoms by boosting an efficient immune response. PEP vaccination was traditionally applied in several infectious diseases like rabies, measles or Hepatitis B (6). PEP is part of the WHO’s strategy to control the COVID-19 pandemic (7). Monoclonal antibodies have previously received FDA approval for PEP, however, recently their use is not recommended as the globally dominant Omicron variant is not susceptible to them. Nevertheless, a retrospective study shows that vaccination with BNT162b2 is effective against COVID-19 related death in PEP setting (8). PEP vaccination would be especially important in the light of reinfection of vaccinated individuals due to waning humoral immunity and decreased neutralizing activity against novel SARS-CoV-2 variants of the presently available vaccines (9).

T cells play a central role in COVID-19 outcome even in the absence of humoral immune response (10–14) and recent data emerge that SARS-CoV-2 T cell response is indispensable to control viral clearance, prevent infection without seroconversion or presence of neutralizing antibodies (15–17) and confer long-term immune memory (18, 19). The role of T cell responses is further inferred from the observation that although the dominant Omicron (B.1.1.529) variant can readily infect vaccinated subjects due to reduced recognition by antibodies (20, 21), T cell immunity is largely unaffected in the vaccinees (22, 23). These observations suggest that T-cell-based vaccines against conserved epitopes of SARS-CoV-2 might be the key to protect against novel VOCs (15).

We developed a novel approach for the selection of immunoprevalent SARS-CoV-2-derived T cell epitopes using an in silico cohort of HLA-genotyped individuals with different ethnicities (24). Based on this tool, nine 30-mer peptides, containing T cell epitopes were selected from the four major structural proteins of SARS-CoV-2 and included in a peptide vaccine candidate, PolyPEPI-SCoV-2. Pre-clinical data in mice showed that PolyPEPI-SCoV-2 generates broad and robust Th1-biased CD4+ and CD8+ T cell responses after subcutaneous injection and vaccine-specific T cells were predominantly present in COVID-19 convalescents’ blood (25).

In this study we have addressed whether a purely T cell vaccine could provide protection against COVID-19 and whether it might be used as a PEP vaccine in SARS-CoV-2 infection. To this end, we evaluated the immunogenicity and protective efficacy of PolyPEPI-SCoV-2 in hamsters that was proven to be an excellent animal model for severe COVID-19 (26–29). Our data show that PolyPEPI-SCoV-2 elicits a strong memory T cell response in vaccinated animals in conjunction with disease amelioration, in both pre- and post-exposure settings. Our data may support the application of T cell-based vaccines in the development of alternative vaccination strategies against symptomatic COVID-19 caused by the novel VOCs.

## 2 Methods

### 2.1 Animals and study design

Animal experiments were approved by the Central Authority for Scientific Procedures on Animals (Centrale Commissie Dierproeven) and conducted in accordance with the European guidelines (EU directive on animal testing 86/609/EEC) and local Dutch legislation on animal experiments. The in-life phase took place at Viroclinics Biosciences B. V., Viroclinics Xplore, Schaijk, the Netherlands. All Viroclinics personnel performing the clinical observations and laboratory analysis supplemented with data interpretation were blinded by allocating a unique sample number to each sample collected and investigated.

**Figure 1:**
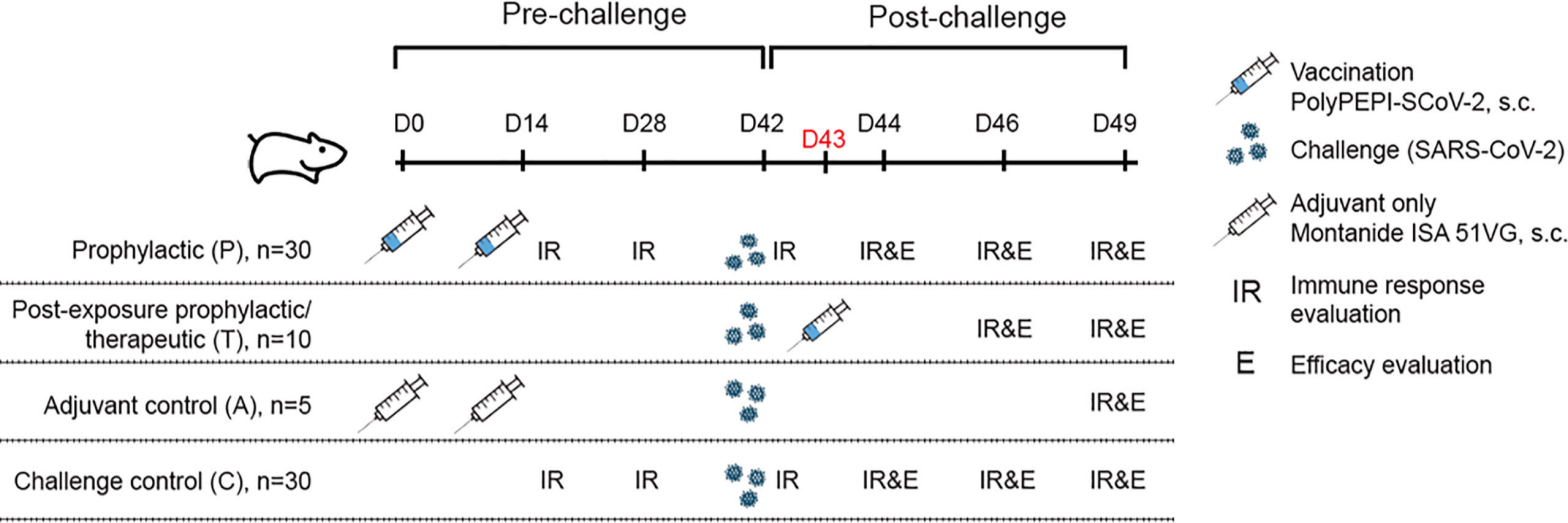
Study outline and overview of experimental groups. 9-10 week old Syrian hamsters (n=75) were divided into 4 cohorts. Hamsters were immunized with PolyPEPI-SCoV-2 subcutaneously on Day 0 (D0) and D14 (prophylactic setting, P) or as a PEP treatment (T) on D43, one day after challenge with SARS-CoV-2. As controls, hamsters were injected with adjuvant only (A) or were left untreated (C). Where indicated, (A, C) controls were combined as Controls (C):( A+C). Hamsters were challenged with 10^2^ TCID50 SARS-CoV-2 on D42 and 5 animals were sacrificed in each group for immune response (T cell response and antibody, IR) and efficacy evaluation (body weight, virology and pathology, (E) at study days (D) indicated on the figure.

75, male, SPF Syrian (golden) hamsters (Mesocricetus auratus), aged 9–10 weeks at the start of the study, were purchased from Janvier Labs (France). Hamsters were immunized subcutaneously (s.c.) with 300 μL vaccine or mock vaccine (150 μL per hind limb) under isoflurane (3-4%/O_2_) anesthesia on Day 0 (D0) and D14. Treatment groups included prophylactically vaccinated animals (P), post-exposure or therapeutic group (T), unvaccinated control without any injection (C), and adjuvant only group receiving Montanide ISA 51 VG adjuvant only, without peptides (mock vaccine, A) (**Figure 1**). On D42, hamsters were intranasally challenged with 10^2^ TCID50 of SARS-CoV-2 (BetaCoV/Munich/BavPat1/2020) equally distributed into each nostrils (50- 50μL). To analyze immunogenicity, lymphoid tissues (spleen and proximal and distal lymph nodes) were collected from n=5 animals of the indicated groups on D14, D28 and D42 and on D44, D46 and D49 after challenge. Additionally, bloods were sampled on the same days from the indicated animals. To measure vaccine efficacy, on the day of infection, prior to challenge, and every second day post infection, 5-5 lung and nasal turbinate tissue samples were collected from animals of the indicated groups under isoflurane anesthesia. Respiratory tissues collected after necropsy were analyzed for viral load and for histopathological changes. Throat swabs were collected from D42 to D49. Further, body weight was measured on D0, D28, D42 and every day after challenge. Throat swabs were collected in virus transport medium, aliquoted, and stored until time of analysis. Tissues were sent directly to ImmunXperts on wet ice. Blood samples from the medial canthus of the eye were collected under isoflurane anesthesia processed for serum isolation, aliquoted and stored frozen until analysis of antibody levels.

### 2.2 PolyPEPI-SCoV-2 vaccine

PolyPEPI-SCoV-2 consists of nine 30-mer peptides derived from SARS-CoV-2 proteins (three from S, four from N, one from E and one from M). Each 30-mer comprises both CD8+ and CD4+ T cell epitopes selected based on multiple autologous HLA allele-binding capacity during in silico design, as described earlier (25). PolyPEPI-SCoV-2 vaccine peptides were manufactured in GMP grade by Ambiopharm, Inc. (North Augusta, SC, USA). The vaccine product (peptide mix) was manufactured by Bioserv Co. (San Diego, CA, USA) in GMP aseptic fill & finish process. Briefly, peptides were dissolved in DMSO (Gaylord Chemical Co., USP grade) to achieve a net concentration of 1 mg/mL/peptide and subsequently diluted with sterile water for injection (Baxter, USP grade) to a final concentration of net 0.2 mg/mL/peptide. The peptide mix solution was filtered through 0.22 µm filter, filled into sterile vials and stored frozen until use. The vaccine quality was verified by confirming the identity and content of the peptides, determining particulate contamination, pH and microbiological quality (bacterial endotoxin and sterility) of the product. Ready-to-inject vaccine preparations were prepared by emulsifying equal volumes of thawed peptide mix solution and Montanide ISA 51 VG adjuvant (Seppic, France) following the standard two-syringe protocol provided by the manufacturer. The mock vaccine (adjuvant only) was prepared using saline (instead of peptide solution) and emulsified with equal volume of Montanide ISA 51 VG, as described above.

The 9-mer and 30-mer test peptides used in the immunological assays were manufactured by Intavis Peptide Services GmbH&Co. KG (Tübingen, Germany) and PEPScan (Lelystad, The Netherlands) using solid-phase peptide synthesis. The 9-mer peptides used for testing the PolyPEPI-SCoV-2-specific CD8+ T cell responses are fragments of the 30-mer vaccine peptides (**Supplementary Table 1**.)

### 2.3 Detection of replication competent virus

Quadruplicate 10-fold serial dilutions were used to determine the virus titers in confluent layers of Vero E6 cells. To this end, serial dilutions of the samples (throat swabs and tissue homogenates) were made and incubated on Vero E6 monolayers for 1 hour at 37°C. After incubation, Vero E6 monolayers were washed and incubated for 4-6 days at 37°C in a CO_2_ thermostat. Plates were scored using the vitality marker WST8 (colorimetric readout, Sigma-Aldrich) according to the manufacturers’ instructions. Briefly, 20 μL per well of WST-8 stock solution (1:5 dilution) was added and incubated 3-5 hours at RT. Subsequently, plates were measured for optical density at 450 nm (OD450) using a micro plate reader and visual results of the positive controls (cytopathic effect, CPE) were used to set the limits of the WST-8 staining (OD value associated with CPE). Viral titers (50% tissue culture infectious dose, TCID50) were calculated using the Spearman-Karber method.

### 2.4 Detection of viral RNA

Throat swabs and homogenized tissue samples were used to detect viral RNA. Briefly, after RNA isolation, Taqman PCR was performed using specific primers (E_Sarbeco_F: ACAGGTACGTTAATAGTTAATAGCGT and E_Sarbeco_R: ATATTGCAGCAGTACGCACACA) and probe (E_Sarbeco_P1: ACACTAGCCATCCTTACTGCGCTTCG) as described by Corman et al. (30) with the TaqMan^®^ Fast Virus 1-Step Master Mix (ThermoFischer Scientific). The number of virus copies in the different samples were calculated and expressed as log10 values.

### 2.5 Virus neutralization (VN) assay

VN assay was performed on blood samples collected during the study. In short, samples were heat inactivated for 30 minutes at 56°C. Subsequently, serial two-fold dilutions of the samples were prepared in infection medium in triplicates in 96-wells plates starting with a dilution of 1:5. The sample dilutions were then incubated with a fixed amount of virus (200 TCID50/well or 4000 TCID50/ml of BetaCoV/Munich/BavPat1/2020 SARS-CoV-2) for 1 hour at 37°C leading to a starting dilution of the serum in the assay of 1:10. Next, the virus-antibody mixtures were transferred onto Vero E6 cell culture monolayers and incubated for 5-6 days at 37°C. Plates were scored based on CPE using the vitality marker WST8 as described above. VN titers were calculated according to the method described by Reed & Muench.

### 2.6 ELISA

Total SARS-CoV-2 Spike (S) and Nucleocapsid (N) -specific IgG in serum were quantified by ELISA. High binding 96 well MaxiSorp Immunoplates (Nunc) were incubated overnight, at 4°C with 100 µl of 0.5 µg/mL of SARS-CoV-2 pre-S (Nexelis, lot: NL2007D-N) or N (Nexelis, lot: NL2008A-N) prepared in PBS. The protein solution was removed, and the plates were washed 3 times with 300 µL of PBS supplemented with 0.05% Tween 20 (PBST). Blocking solution (PBST containing 5% non-fat dried milk) was added at 200 µL per well and the plates were incubated for 1 hour at 37°C. A pool of sera from SARS-CoV-2 pre-S or N-immunized hamsters was diluted in blocking solution as appropriate and served as a standard. Positive and negative control sera were diluted 1/50 in blocking solution. Serum samples were diluted at 1/50 in blocking solution followed by seven 2-fold serial dilutions in blocking solution. Diluted serum samples and controls were incubated on the plate for 2 hours at 37°C. The plates were washed 3 times with 300 µL of PBST. Following the last wash step, 100 µL of HRP-conjugated goat anti-hamster IgG heavy and light chain antibody (Bethyl, Cat: A140-201P, diluted 1/12500 in blocking solution) was added to each well. The plates were incubated for 1 hour at 37°C and then washed 5 times with 300 µL PBST. TMB substrate (BioRad) was added to each well and the plates were incubated at RT for 30 minutes. The colorimetric reaction was stopped by addition of 100 µL/well 0.36 N sulfuric acid. Absorbance at 450 nm was measured by spectrophotometry using a SpectraMax ID3 microplate reader and results analyzed using SoftMax Pro 7.1 (Molecular Devices, San Jose, CA). Limit of detection was defined as 20% of standard curve.

### 2.7 Ex vivo ELISpot

Ex vivo ELISpot assays to measure the number of IFN-γ secreting cells was performed by ImmunXperts (Q2 Solutions; Belgium) from lymph node and spleen samples as follows: IFN-γ hamster ELISpot(Mabtech AB, Sweden) plates were blocked with RPMI medium containing 10% FBS, then peptides (5 μg/mL final concentration) or peptide pools (5 μg/mL per peptide final concentration) were added to the relevant wells. 200,000 or 400,000 spleen or lymph node cells/well were plated in triplicate (stimulation conditions) or 6-plicates (reference conditions) and incubated overnight at 37°C, in a 5% CO_2_ incubator before development. Development of the ELISpot plates was performed according to the manufacturer’s recommendations. After removing cells, detection antibodies diluted in PBS containing 0.5% Fetal Bovine Serum were added to the wells and the ELISpot plates were incubated for 2 hours at RT. After washing, streptavidin-ALP diluted in PBS containing 0,5% Fetal Bovine Serum was added and incubated for 1 hour. After washing, ready to use and filtered substrate solution (BCIP-NBT plus) was added until distinct spots occurred. Before readout using the Mabtech IRIS™ automated ELISpot/FluoroSpot reader, the ELISpot plates were extensively washed and treated with 4% paraformaldehyde followed by washes and dried at RT for 24 hours protected from light. All data were acquired with a Mabtech IRIS™ reader and analyzed using Mabtech Apex TM software. Unstimulated (DMSO) negative control and SEB and PMA/ionomycin positive control were included as assay controls. Results are illustrated as spot forming units (SFUs) minus the background DMSO control per 10^6^ PBMCs. Ex vivo ELISpot results were considered positive when the test value was at least two-times higher than DMSO negative control after subtracting non-stimulated control.

### 2.8 Histopathology

Histopathology was assessed by pathologists from the University of Veterinary Medicine (Hannover, Germany). Gross pathology was assessed by a pathologist from the Utrecht University (Utrecht, The Netherlands) (histopathology and gross pathology were evaluated by different pathologists in different part of the isolated lung tissue.) On D44, D46, D49 animals were autopsied by opening the thoracic and abdominal cavities and lung tissue isolated. The extent of pulmonary consolidation was assessed based on visual estimation of the percentage of affected lung tissue (gross pathology) on all lung lobes. The left half of lung tissue was collected and preserved in 10% formalin for histopathological examination and analysis by immunohistochemistry. Lungs were routinely processed, paraffin wax embedded, micro-sectioned to 3 μm on glass slides, and stained with haematoxylin and eosin (H&E) for histopathological evaluation. The H&E-stained tissue sections were examined by light microscopy for histopathology scoring, as well as for the presence of any other lesions. Representative images of lung lesions from each group were taken using an Olympus, VS200 digital slide scanner (Olympus Deutschland GmbH, Hamburg, Germany). To assess disease severity in the lung, each entire slide was examined and scored for the presence or absence of alveolar edema, alveolar hemorrhage, and type II pneumocyte hyperplasia (0= no, 1= yes). The degree and severity of inflammatory cell infiltration and damage in alveoli, bronchi/bronchioles were scored for alveolitis and bronchitis/bronchiolitis: 0= no inflammatory cells, 1= few inflammatory cells, 2= moderate number of inflammatory cells, 3= many inflammatory cells. Extent of peribronchial/perivascular cuffing: 0= none, 1 = 1–2 cells thick, 2 = 3–10 cells thick, 3= over 10 cells thick. Additionally, the extent of alveolitis/alveolar damage was scored per slide: 0 = 0%, 1 ≤ 25%, 2 = 25–50%, 3≥50% (29). The cumulative score (sum) for the above parameters provided the total lung score with a possible maximum score of 18. The following histopathology parameters were included: alveolitis, alveolar damage, alveolar edema, alveolar hemorrhage, type II pneumocyte hyperplasia, bronchitis, bronchiolitis, peribronchial and perivascular cuffing.

### 2.9 Statistical analysis

Statistical differences between the control and vaccinated groups were evaluated by one-tailed Mann-Whitney U-test in case of IFN-γ ELISpot, ELISA, neutralizing titers as measured by VN assay, viral load as measured by TCID50, histopathology scores and body weight loss. Statistical analyses were performed using GraphPad Prism software, version 9.3.1. Data were considered statistically significant in case of p<0.05 (α=0.05). Results of statistical analysis are illustrated as follows: p>0.05 non-significant (ns), p<0.05 significant (*), p<0.01 significant (**), p<0.001 significant (***) as assessed by one-tailed Mann-Whitney test.

## 3 Results

### 3.1 PolyPEPI-SCoV-2 induces rapid T cell response against SARS-CoV-2 infection

To assess the induction of immune responses and the efficacy of PolyPEPI-SCoV-2 *in vivo*, 9-10 week old Syrian hamsters (n=75) were vaccinated subcutaneously in a prophylactic setting (P) on Day 0 (D0) and D14, and in a PEP/therapeutic setting (T) on D43, one day after challenge with SARS-CoV-2 (**Figure 1**). Two control cohorts were applied: an unvaccinated control (C) and a mock vaccinated group receiving Montanide ISA 51 VG adjuvant only without the PolyPEPI-SCoV-2 peptides (A) (see details in Materials and Methods and **Figure 1**). All animals were challenged intranasally with 10^2^ TCID50 SARS-CoV-2 virus on D42. The immune response was investigated on the indicated days *via* IFN-γ secretion of T cells in both the spleen and lymph nodes (LNs) and antibody measurements alongside with evaluation of efficacy *via* body weight monitoring, virology and pathology assessments (**Figure 1**).

**Figure 2:**
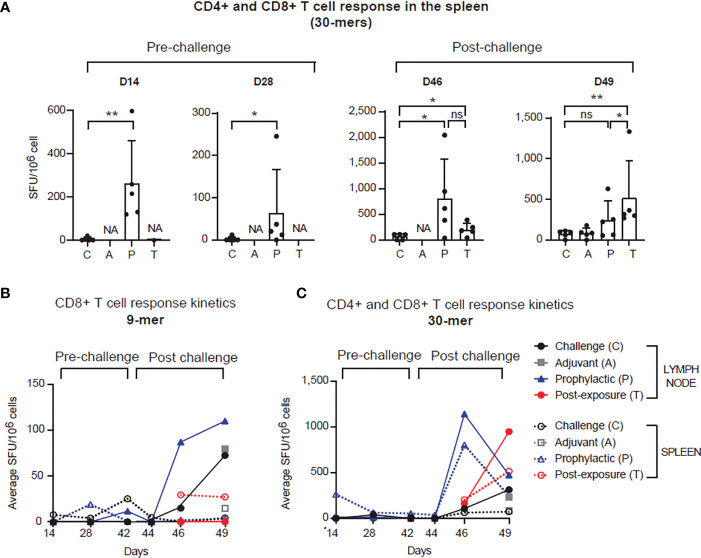
PolyPEPI-SCoV-2 induces strong and rapid adaptive T cell immunity pre- and post-infection. **(A)** PolyPEPI-SCoV-2 induces adaptive T cell immunity in the spleen. Immunogenicity is illustrated on D14, D28, D46 and D49 as mean ± SD spot forming unit (SFU) per 10^6^ spleen cells of 5 animals against the 30-mer peptide pool (one-tailed Mann-Whitney test, ns, p>0.05, (*) p<0.05, (**) p<0.01. NA, not-analyzed (response not investigated). **(B, C)** T cell response kinetics was evaluated on D14, D28, D42 (0 d.p.i.), D44 (2 d.p.i.), D46 (4 d.p.i.) and D49 (7 d.p.i.) against **(B)** the 9-mer and **(C)** the 30-mer peptide pools (S, N, M and E peptides pooled together). Data are shown as average SFU per 10^6^ spleen cells of 5 animals or SFU per 10^6^ pooled lymph node cells (pool of 5 animals per group). Response to 9-mer represents mainly CD8+ T cell while response to 30-mer depicts combined CD4+ plus CD8+ T cell activation.

Immunization with PolyPEPI-SCoV-2 led to activation of cellular immunity in Syrian hamsters, confirming previous data obtained in mice (25) (**Figure 2A**, left and **Supplementary Figure 1**). Significantly elevated T cell responses were observed in the spleen of vaccinated animals two weeks after both the first (p<0.01) and the second dose (p<0.05), compared to unvaccinated controls (**Figure 2A**, left). Challenge with SARS-CoV-2 resulted in elevated T cell responses in all tested cohorts compared to pre-challenge phase (**Figure 2A**, right). On D46, four days post infection (d.p.i), T cell responses were much higher both in magnitude and in response rate in the vaccinated animals (P) than in the challenged control animals (at least 4-fold increase, p<0.05), indicating the rapid activation of memory T cells that were elicited by vaccination. Interestingly, a single vaccine dose administered one day after virus challenge (T group) also resulted in significantly higher T cell response on both D46 (p<0.05) and D49 (p<0.01), compared to challenge-only, unvaccinated animals (C group) (**Figure 2A**, right). Importantly, PolyPEPI-SCoV-2 induced diverse T cell responses against all four structural proteins of SARS-CoV-2 (**Supplementary Figures 1B, C**). The diversity of T cell responses was maintained after viral-challenge as well, albeit dominated by S-specific responses (**Supplementary Figures 1B, C**).

Pre-challenge T cell responses were less detectable in the lymph nodes (**Figures 2B, C** and **Supplementary Figure 1A**). In contrast, the recall response against 9-mers (depicting mainly CD8+ T cell response) after viral challenge was elevated both in the P and C groups but with different kinetics (**Figure 2B**). A robust T cell response was seen earlier (on D46) for the vaccinated (P) than for the control group (C), the latter reaching similar level only on D49 (7 d.p.i.). In the spleen, CD8+ T cell responses were detected only for the T group on D46 (4 d.p.i.) and maintained by D49 (7 d.p.i.), as well. T cell response kinetics against the 30-mers (likely mixed CD8+ and CD4+ T cells) for the vaccinated groups (P and T), showed a fast increase both in the spleen and LNs peaking by D46 (4 d.p.i.) for P and by D49 (7 d.p.i.) for T (**Figure 2C**). For control animals this was slower and was present at a significantly lower level (**Figures 2B, C**). These data indicate that PolyPEPI-SCoV-2 elicits a diverse pool of SARS-CoV-2-specific effector and memory T cells enabling rapid and robust T cell response against the infection in both prophylactic and PEP settings.

### 3.2 PolyPEPI-SCoV-2 does not elicit antibody production

**Figure 3:**
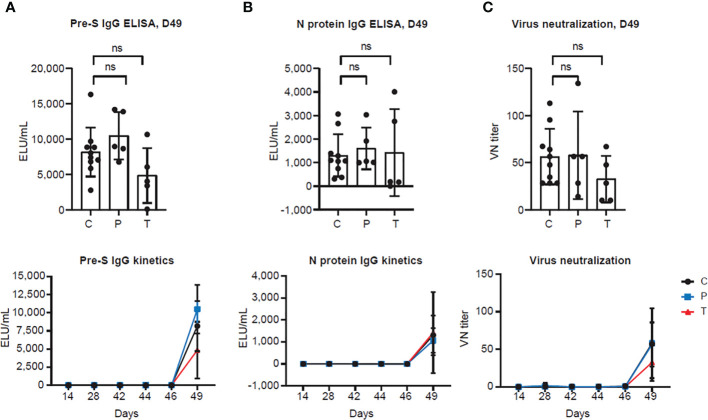
PolyPEPI-SCoV-2 does not induce antibody secretion and virus neutralization **(A, B)** Antibody response measured by **(A)** pre-S and **(B)** N protein IgG ELISAs from hamster sera. Results are shown as average of secreted antibodies (ELU/mL) ± SD of 5 animals per group (or 10 in the Controls group) measured in duplicates from D14 to D49 (lower panel) and separately illustrated on D49 (upper panel). **(C)** SARS-CoV-2 neutralization titers were measured by virus neutralization assay. Results are shown as average of virus neutralizing (VN) titer ± SD of 5 animals per group (or 10 in the Controls group) measured in triplicates from D14 to D49 (lower panel) or separately illustrated on D49 (upper panel). **(C)**: Controls (combined challenge and adjuvant controls), P: prophylactic, T: post-exposure prophylactic. ns, non-significant result, p>0.05 by one-tailed Mann-Whitney test.

In the next step, we investigated whether PolyPEPI-SCoV-2 induces secretion of SARS-CoV-2 specific antibodies. In contrast to the BALB/c and humanized mouse models where previously elevated total IgG response was measured after administration of PolyPEPI-SCoV-2 (25), no SARS-CoV-2-specific antibody secretion could be detected neither in the control nor in the vaccinated hamsters until D49 (**Figure 3**). Although a strong anti-pre-S and anti-N level antibody secretion could be observed one week after viral challenge, PolyPEPI-SCoV-2 did not cause further elevation in the challenge-induced antibody level indicating that the vaccine itself does not induce virus-specific IgG production (**Figures 3A, B**). The same tendency could be observed in case of virus neutralization: all SARS-CoV-2 challenged hamsters showed neutralizing activity on D49, with no difference in the vaccinated groups indicating the absence of vaccine-induced neutralizing antibodies (**Figure 3C**). These results suggest that although PolyPEPI-SCoV-2 induces a strong T cell response, it does not activate humoral immunity and secretion of SARS-CoV-2-specific antibodies.

### 3.3 Evaluation of the protective effect of PolyPEPI-SCoV-2

**Figure 4:**
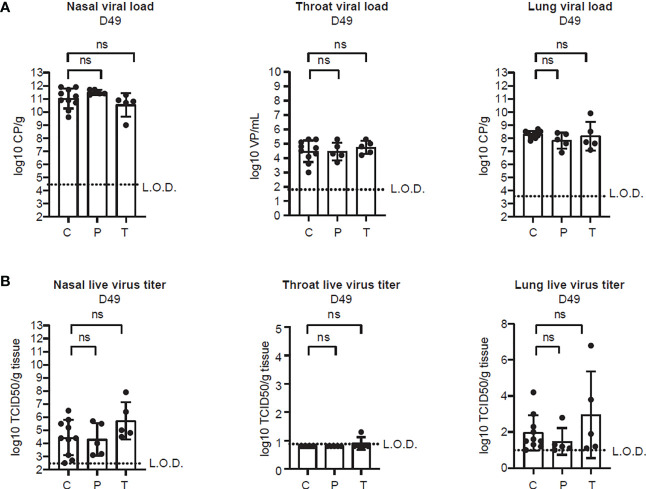
PolyPEPI-SCoV-2 does not alter viral clearance from the respiratory tract. **(A)** Viral load in the lung tissue, nasal turbinates and throat, determined on D49 by PCR. Results are shown as average of log10 VP/mL or log10 CP/mL ± SD of 5 animals per group (or 10 in the Controls group). **(B)** Replication-competent virus isolated from the lung, nasal turbinates and throat, measured by TCID50 assay on D49. Results are shown as average of log10 TCID50/gram tissue ± SD of 5 animals per group (or 10 in the Controls group). Dotted lines indicate the limit of detection (LOD). CP, crossing-point value, TCID50/g: 50% tissue culture infective dose per gram tissue, VP, virus particles, C, Controls (combined challenge and adjuvant controls), P: prophylactic, T: post-exposure prophylactic. ns, non-significant result, p>0.05 by one-tailed Mann-Whitney test.

One month after the second immunization (on D42), hamsters were challenged with 10^2^ TCID50 of SARS-CoV-2 virus. Groups of animals were sacrificed on 2, 4, and 7 d.p.i. for viral load and histopathology analyses in the various organs of the upper- and lower respiratory tracts. As indicated in **Figure 4**, SARS-CoV-2 challenge allowed breakthrough infections both in the upper- and lower respiratory tracts but no difference in the viral load (**Figure 4A**) and in the live virus titer (**Figure 4B**) could be observed between the vaccinated (P or T) and control (C) animals. The viral nucleic acid was present through the whole observation period after challenge (D44 - D49) and it did not decrease below the detection limit (**Figure 4A** and **Supplementary Figure 2**). On the contrary, the live virus titer dropped in all cohorts on D49 but was undetectable only in the throat (**Figure 4B** and **Supplementary Figure 3**).

**Figure 5:**
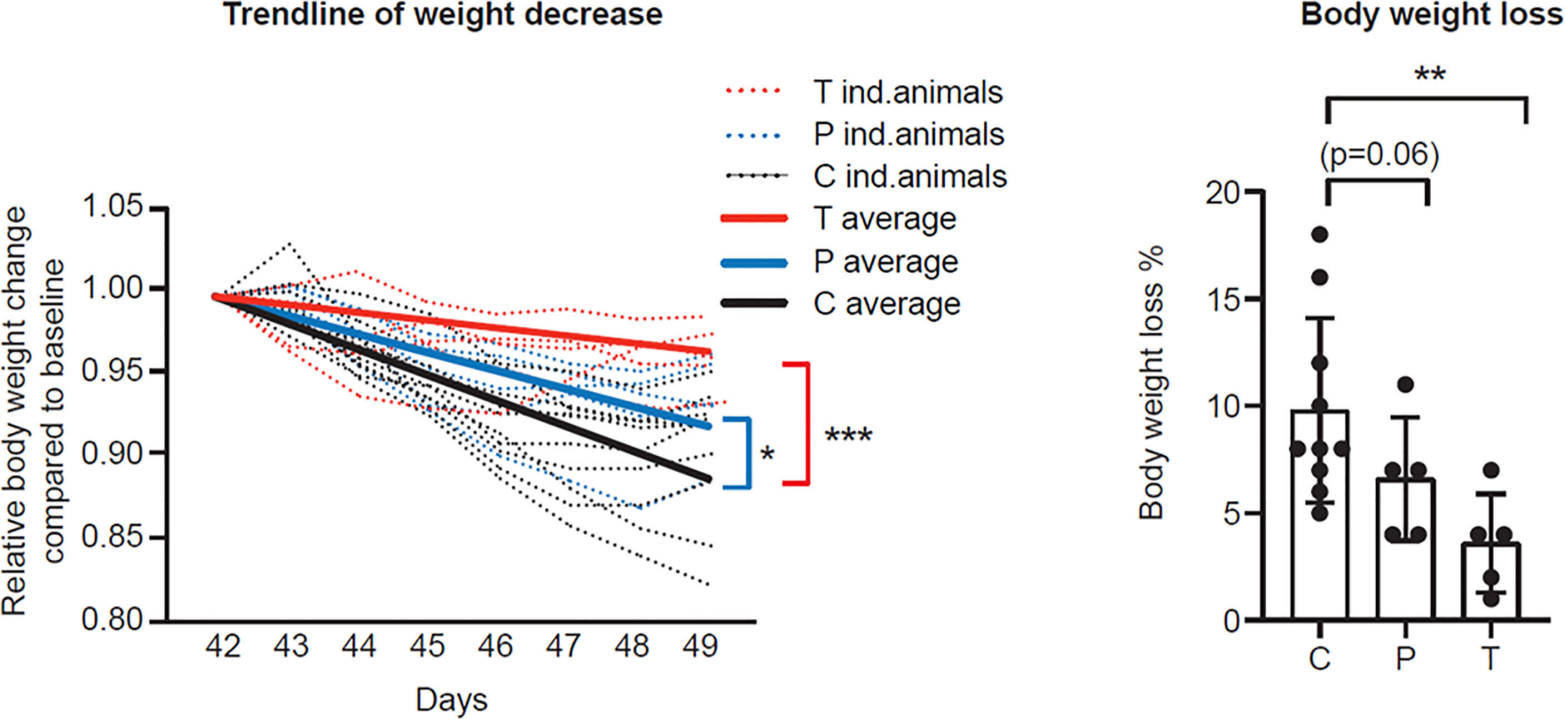
PolyPEPI-SCoV-2 decreases body weight loss of SARS-CoV-2 infected hamsters. Post challenge daily relative body weight changes are represented for each individual animal (left panel) or on D49 (right panel) compared to D42. Results are shown as average of body weight loss % ± SD of 5 animals (10 Controls) per group (right panel). Solid lines represent average relative weight loss trendlines for the cohorts (left panel). C, Controls (combined challenge and adjuvant controls), P: prophylactic, T: post-exposure prophylactic. *p<0.5, **p<0.01, ***p<0.001 by one-tailed Mann-Whitney test.

To assess the potential disease-modifying effect of vaccinations, body weights were monitored daily for each animal up to D49 (7 d.p.i.). Unvaccinated and adjuvant-only animals behaved similarly in terms of post-challenge weight loss (**Supplementary Figure 4A**, D49), i.e. the adjuvant had no acute effect, therefore in further analysis these animals were combined as Controls (C+A). All animals survived until the end of the study, however body weights showed a continuously decreasing trend which was more intense for the Control animals (C+A) compared to both the T (p<0.001) and the P (p<0.05) groups (**Figure 5**, left panel). On D49, prophylactically vaccinated animals (P) showed a more moderate weight loss compared to the control group (average weight loss 6.6% ± 2.9 vs. 9.8% ± 4.3, respectively, p=0.06). Similarly, post-exposure application of PolyPEPI-SCoV-2 (T) significantly decreased the disease induced body weight loss compared to the Control group (3.6% ± 2.3 vs. 9.8% ± 4.3, p<0.01) (**Figure 5**, right panel).

**Figure 6:**
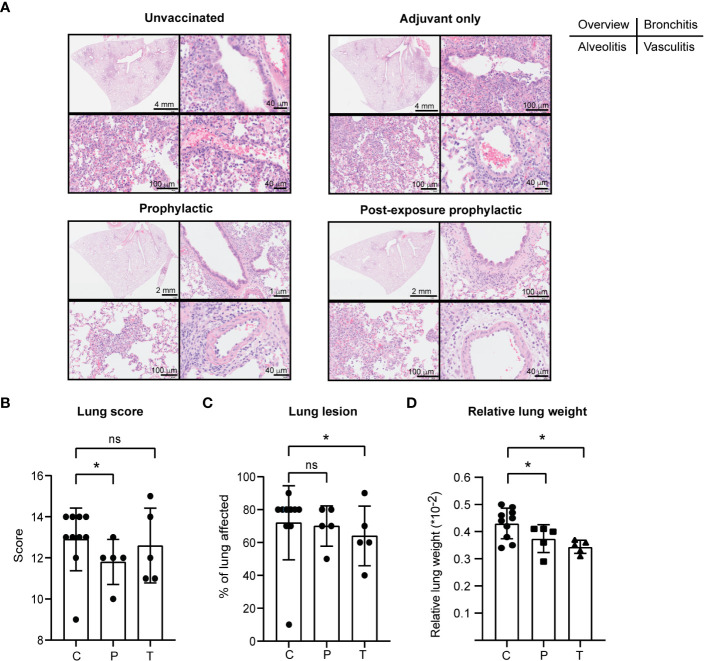
PolyPEPI-SCoV-2 decreases lung pathology of SARS-CoV-2 infected hamsters. **(A)** Lung scores (see subpanel B) were evaluated based on hematoxylin-eosin (H&E) stained tissue sections, examined by light microscopy. Representative results of one animal from each group on D49 are shown as follows: overview of the H&E stained lung (upper left panels), bronchitis (upper right panels), alveolitis (lower left panels), vasculitis (lower right panels). **(B–D)** Lung pathology was evaluated as lung score **(B)**, lung lesions **(C)** and relative lung weight **(D)** on D49. Data are presented as sum of the score of the disease parameters (detailed in Materials and Methods and in **Supplementary Figure 5**). **(C)** Gross pathology was evaluated on the whole lung lobes *via* visual observation by an independent pathologist and results provided as the % of lung area affected (lung lesion). **(D)** Relative lung weight illustrated as percentages of body weight [(lung weight/body weight)*100]. Results are shown as average ± SD of 5 animals (10 Controls) per group. C, challenge and adjuvant controls, P, prophylactic, T, post-exposure prophylactic. ns, p>0.05, (*) p<0.05 calculated using one-tailed Mann-Whitney test.

Further, PolyPEPI-SCoV-2 exerted a positive protective effect on lung pathology caused by SARS-CoV-2 infection. Despite the relatively low dose of SARS-CoV-2 challenge, the infection caused severe disease in both the upper- and lower respiratory tracts. The number and severity of symptoms increased from D44 to D49 as assessed by histopathology and gross pathology of the lung tissues (**Supplementary Figure 4** and **Supplementary Figure 5**). No spontaneous remission was observed. Lung scores were evaluated by histopathology. Representative results of one animal from each group are shown on **Figure 6A** as follows: overview of the H&E stained lung (upper left panels), bronchitis (upper right panels), alveolitis (lower left panels), vasculitis (lower right panels). By D49, in the control group (C+A), each of the main histopathologic parameters evaluated (7/7, alveolar oedema absent, not included) indicated a severe disease (score 2 or 3), whereas in the vaccinated group (P+T) only three of them had severe disease by all parameters and for seven animals 1-3 parameters indicated mild or no symptoms (score 0 or 1) (**Supplementary Figures 4B**, **5**). Accordingly, the total lung score (indicating alveolar damage, haemorrhage, oedema, alveolitis, bronchiolitis, bronchitis and vasculitis) was significantly decreased in the P group (p<0.05) and showed a tendency toward reduction in the T group (**Figures 6A, B**). In addition, gross pathology performed by an independent pathologist indicated for both vaccinated groups decreased area affected by lesions (representing inflammation and infiltration of immune cells) compared to control animals, but the differences reached statistical significance only for the T group (p<0.05, **Figure 6C**). This was supported by the relative lung weights which were significantly lower in the vaccine-treated hamsters both in case of prophylactic as well as PEP applications (p<0.05) (**Figure 6D**). Important to mention that neither the total lung score (histopathology) nor the gross pathology suggested enhancement of the disease in the vaccinated animals indicating that PolyPEPI-SCoV-2 does not aggravate lung disease (a typical sign of vaccine-associated enhanced disease, VAED) in challenged hamsters.

These results indicate that PolyPEPI-SCoV-2-induced T cell immunity can reduce the clinical signs and severity of COVID-19 despite being not able to alter the viral clearance.

### 3.4 Impact of SARS-CoV-2 variants on PolyPEPI-SCoV-2

**Figure 7:**
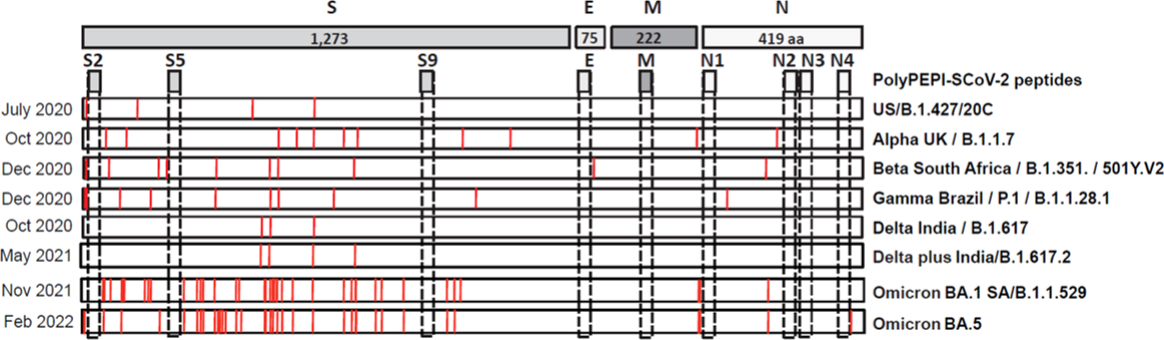
Impact of SARS-CoV-2 variants of concern (VOC) on PolyPEPI-SCoV-2 peptides. Mutations described for major VOCs are shown and compared to PolyPEPI-SCoV-2 peptides (S2, S5, S9, E, M, N1, N2, N3 and N4) (25).

Due to the high mutational rate, SARS-CoV-2 is continuously changing and currently, WHO declares five major variants of concern (VOC) – B.1.1.7-Alpha, B.1.351-Beta, P.1-Gamma, B.1.617.2-Delta, B.1.1.529-Omicron, the last became highly dominant with sub-lineages BA.1 to the most recent BA.4 and BA.5 (the latter ones are almost identical in both quality and quantity of mutations) (31). VOCs that are able to escape vaccine-induced immunity urges the need for designing vaccines against invariable part of the virus and has shifted the focus to T cell vaccines (15). To investigate the impact of SARS-CoV-2 VOCs on PolyPEPI-SCoV-2, the amino acid sequences of vaccine peptides were compared to the corresponding mutated regions in each VOC. As illustrated in **Figure 7**, PolyPEPI-SCoV-2 was affected only by a single amino acid change in the N4 peptide by the newest Omicron BA.5 sub-lineage indicating broad coverage of vaccine peptides for SARS-CoV-2 variants.

## 4 Discussion

T cell immunity has been shown to protect and combat early infection of SARS-CoV-2 and provide long-standing immune memory (14, 32) in contrast to neutralizing antibody levels that wane significantly by 6-8 months after the vaccination or natural infection (33). Consequently, several T-cell based peptide vaccines have been developed and tested in pre-clinical (34–38), or clinical trials (30, 39–41). Previously, PolyPEPI-SCoV-2 induced broad immune responses in humanized mice and peptide specific T cells were also identified in the blood of convalescent subjects (25). However, mice are less suitable model to study severe COVID-19 (26) and appropriate mouse models have been developed only recently (42) after conduct of our experiments. Hence, in this study, PolyPEPI-SCoV-2 was evaluated for T cell immunogenicity and protective efficacy against COVID-19 in a hamster model of severe COVID-19 (29).

To our best knowledge, no data have been published so far about SARS-CoV-2 vaccine-induced T cell immunity in hamsters as well as no kinetics of the response was generally studied, which enables collection of direct evidences for the role of T cells in amelioration of symptomatic COVID-19. PolyPEPI-SCoV-2 elicited rapid, multi-antigenic CD4+ and CD8+ memory T cell responses, which were able to recognize multiple target peptides on SARS-CoV-2-infected cells. The recall T cell response was fast and strongly elevated, preceding with several days both the cellular and humoral immune reaction of unvaccinated animals. This T cell response was directed mainly against the S protein, especially after viral challenge which can be explained by the higher number of CD4+ T cell epitopes and more efficient presentation of S peptides on MHCII which has been shown to directly support and correlate with cytotoxic T cell activity (43) These results indicate that PolyPEPI-SCoV-2 generates a pool of memory cells that can be quickly mobilized upon infection, leading to amelioration of disease severity. The post-exposure application of PolyPEPI-SCoV-2 also led to an increase in the magnitude of T cell immunity, however reached a peak slower (by D49) than prophylactically vaccinated animals indicating activation of the primary immune response. As opposed to virus-induced T cells, in vaccinated animals, T cell responses were detected both in the spleen and lymph nodes. As the novel paradigm identifies the spleen as the primary site for induction of long-lived memory T cell precursors in respiratory viral infections, our observation emphasizes the importance of booster vaccinations in generation of long-lasting immune memory (44).

Although PolyPEPI-SCoV-2 was not sufficient to decrease the viral load in the respiratory tract of infected animals, it could ameliorate the severity of the disease. The benefit of PolyPEPI-SCoV-2 induced T cell responses could be observed in the limited extension of tissue damages, lower level of inflammation in the lung, and the reduced weight loss in the vaccinated groups compared to the control animals supporting the recent results of Kingstad-Bakke et al. publishing T cell conferred protection in the absence of serum neutralizing antibodies (15). Similar results were reported by Pardieck et al. showing that a single T cell epitope of the Spike protein confers protection against lethal SARS-CoV-2 infection after 3 injections (38). Indeed, according to the current view, while humoral immunity and virus neutralizing antibodies are protective against SARS-CoV-2 infection and mild COVID-19, T cells provide protection mainly against severe disease and hospitalization (45). This is strongly supported by our results showing that although PolyPEPI-SCoV-2 was not sufficient to decrease the viral load in the respiratory tract of infected animals, it could ameliorate the disease severity. This is in agreement with data published on other T cell-based peptide vaccines where - without any antibody formation – no complete protection from COVID-19 could be observed (34, 39). On the contrary, in the absence of neutralizing antibodies, CD4+ and CD8+ T cells are able to cooperatively control the disease (15). The reason behind incomplete disease management and lack of viral clearance is probably the absence of vaccine induced antibody production and less likely vaccine-induced tolerogenic mechanisms proved by the shift towards Th1-type response in BALB/c and humanized mice with minimal detection of Th2 cytokines and IL-10 (25).

Here we also provide the first preclinical evidence on potential efficacy of a T cell-based vaccine as PEP in COVID-19. Immunization with PolyPEPI-SCoV-2 one day after viral challenge reduced the disease severity and maintained the general welfare of vaccinated animals likely due to the induction of a broad and robust naïve T cell response that overcame the one induced by natural infection. These results suggest that even in acute infection, T cells are able to contribute to better disease control, probably *via* stimulation of the interferon response (40) and early mobilization compared to virus-specific, high-affinity humoral antibodies (14). Indeed, our result suggests that vaccination-boosted T cells appear earlier than the virus-specific IgG response of which induction require T cell support. It was also confirmed in human subjects that SARS-CoV-2 exposure might induce specific T cell responses without seroconversion and IgG induction (46).

There are a number of limitations in our study that could lead to incomplete disease control. Firstly, PolyPEPI-SCoV-2 was optimized for human HLAs by in silico selection (25), therefore it is likely that the peptide processing, presentation and MHC restriction is suboptimal and highly variable in the outbred Syrian hamsters. Secondly, to improve immunogenicity of PolyPEPI-SCoV-2, Montanide ISA 51 VG was used as an adjuvant that skew the immune response towards a Th1 phenotype without significant induction of humoral immunity. Using this adjuvant however, has the advantage to limit Th2-biased VAED, induce strong T cell response (39), and the adjuvant itself has no therapeutic effect (47, 48). Thirdly, as suggested and proved by Padrieck et al., 2 immunizations might not have been enough to provide complete protection against challenge (38). Further experiments with 3 or more immunizations in unvaccinated or pre-vaccinated animals are necessary to investigate this hypothesis. Last but not least, hamsters experience spontaneous viral load reduction one week after sublethal SARS-CoV-2 challenge (29), limiting the time period where the effect of post-exposure prophylactic vaccines could be monitored (ie. within 7 days in our experiments). Despite these drawbacks, the consistent induction of rapid T cell memory response and the fast proliferation of boosted effector T cells observed in both pre- and post-exposure prophylactic settings might support our findings on the disease-modifying role of T cells in COVID-19 which might be further enhanced by humoral responses. Our results strongly strengthen the initiative that SARS-CoV-2 exposed individuals should be vaccinated to prevent severe disease terminating in death or hospitalization (7, 8, 49). In our hamster challenge study, the infecting virus was the original, Wuhan strain (Munich/BavPat1/2020), however the in silico analysis suggests that PolyPEPI-SCoV-2 could potentially induce resistant immunity against novel VOCs of SARS-CoV-2 as well, since the induced T cells are specific for conserved, universal SARS-CoV-2 specific sequences that are robust along the whole evolution of the virus, so far. However, this remains to be demonstrated in further challenge studies.

Based on the observed rapid and broad T cell response and on the mutation resistance of PolyPEPI-SCoV-2 peptides, one can hypothesize that PEP vaccination might induce cross-reactive cellular immunity and could restore the adaptive immune response of individuals vaccinated against the original SARS-CoV-2 strain. Therefore, the administration of variant-resistant SARS-CoV-2 vaccines either as a complementary booster vaccine or as a PEP injection could spare the manufacturing of new variant-specific vaccines and provide a fast, feasible and long-term solution for the pandemic.

## Non-standard abbreviations

A, Adjuvant-only (mock vaccine) receiving control group in the challenge study; C, Unvaccinated negative control group in the challenge study or combined control group of unvaccinated and adjuvant-only groups (A+C), where indicated; CPE, Cytopathic effect; D0-D49, Study in-life phase length in days, vaccination starts at day 0; E, Envelope protein of SARS-CoV-2; M, Matrix protein of SARS-CoV-2; N, Nucleocapsid of SARS-CoV-2; P, Prophlylactic setting in the challenge study; PolyPEPI-SCov-2, vaccine administered at D0 and D14; PEP, Post-exposure prophylaxis; PolyPEPI-SCoV-2, Nine 30-mer peptides comprising vaccine candidate against SARS-CoV-2 and COVID-19. Comprising peptides are abbreviated by the source/target structure proteins: S2, S5, S9, E, M, N1, N2, N3 and N4; S, Spike protein of SARS-CoV-2; T, Therapeutic or post-exposure setting in the challenge study; TCID50, 50% Tissue Culture Infectious Dose

## Data availability statement

The original contributions presented in the study are included in the article/**Supplementary Material**. Further inquiries can be directed to the corresponding author.

## Ethics statement

The animal study was reviewed and approved by Central Authority for Scientific Procedures on Animals (Centrale Commissie Dierproeven).

## Author contributions

ET, ES, MK, LW, and SP designed the experiments and analyzed the data; ET, ES, and MK wrote the paper. ET, ES, ZC, LM, JT, and OL designed the vaccine. MK, JT, and LM performed the statistical analyses. AB and WR performed histopathology. All authors contributed to the article and approved the submitted version.
