# Supplementary material for: T cell immunity ameliorates COVID-19 disease severity and provides post-exposure prophylaxis after peptide-vaccination, in Syrian hamsters
Source: Front Immunol. 2023 Jan 24;14:1111629. doi: 10.3389/fimmu.2023.1111629 (PMC9902696; doi:10.3389/fimmu.2023.1111629)
Supplement: Supplementary file 1 [file DataSheet_1.docx]

**SUPPLEMENTARY FIGURES AND FIGURE LEGENDS**

**Supplementary Figure 1.**

**
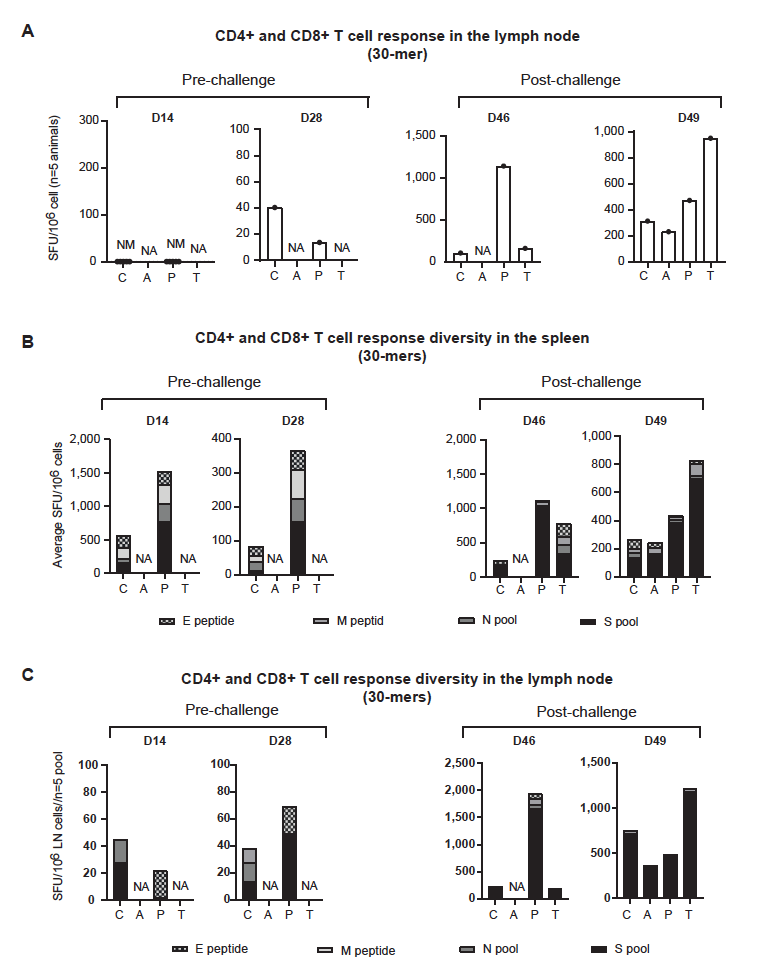
**

Supplementary Figure 1. **PolyPEPI-SCoV-2 increases the diversity and magnitude of T cell immune response**

T cell response and diversity measured ex vivo by IFN-γ ELISpot from pooled lymph node cells of 5 animals. (**A**) Immunogenicity is illustrated on D14, D28, D46 and D49 as spot forming unit (SFU) per 10^6^ pooled LN cells against the 30-mer peptide pool.

**(B)** T cell response and diversity was evaluated against the 30-mer E, M peptides or the N and S peptide pools. Results are shown as average SFU per 10^6^ spleen cells of n=5 animals specific for S pool (black), N pool (dark grey), M (light grey) and E (patterned) peptides.

(**C**) T cell response diversity was evaluated on D14, D28, D46 and D49 against the 30-mer E, M peptides or the N and S peptide pools. Results are shown as SFU per 10^6^ pooled LN cells specific for S pool (black), N pool (dark grey), M (light grey) and E (patterned) peptides from D14 to D49. NA: not-analyzed, NM: non-measurable.

**Supplementary Figure 2.**

**
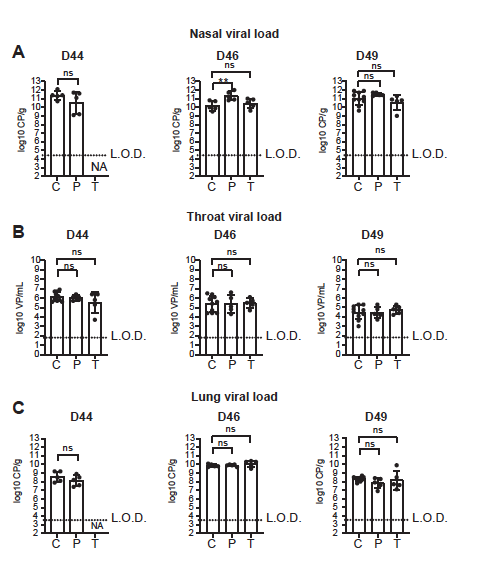
**

Supplementary Figure 2. **PolyPEPI-SCoV-2 does not alter viral load of the respiratory tract**

Syrian hamsters were s.c. immunized with a two-dose regimen of PolyPEPI-SCoV-2 peptide vaccine. Hamsters were challenged intranasally with 10^2^ TCID50 SARS-CoV-2 on Day 42. Viral load was measured on D44, D46, D49 (lung (**C**) and nasal turbinates (**A**) or daily (throat swabs (**B**)) by PCR. Results are shown as average of log10 VP/mL or log10 CP/mL ± SD of 5 animals per group (or 10 in the Controls group) (ns: non-significant result, one-tailed Mann-Whitney test).

Dotted lines indicate the limit of detection (LOD). Data points of challenge and adjuvant groups were combined and are represented as Controls. CP crossing-point value, LOD limit of detection, VP virus particles, C: challenge and adjuvant controls, P: prophylactic, T: therapeutic, ie. post-exposure prophylactic.

**Supplementary Figure 3.**

**
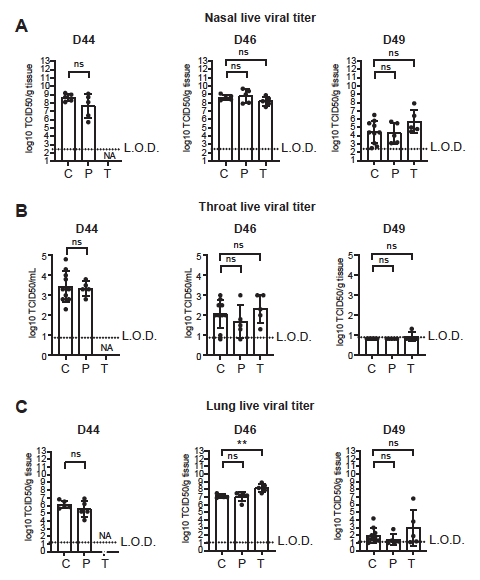
**

Supplementary Figure 3. **PolyPEPI-SCoV-2 does not decrease viral replication in the respiratory tract.**

Syrian hamsters were subcutaneously immunized with a two-dose regimen of PolyPEPI-SCoV-2 peptide vaccine. Hamsters were challenged intranasally with 10^2^ TCID50 SARS-CoV-2 on Day 42. Live virus titer was measured on D44, D46, D49 (lung (**C**) and nasal turbinates (**A**) or daily (throat swabs (**B**)) by TCID50 assay. Results are shown as average of log10 TCID50/gram tissue ± SD of 5 animals per group (or 10 in the Controls group) (ns: p>0.05 non-significant result, **: p<0.01) by one-tailed Mann-Whitney test). Dotted lines indicate the limit of detection (LOD).

LOD limit of detection, TCID50/g 50% tissue culture infective dose per gram tissue, C: challenge and adjuvant controls, P: prophylactic, T: therapeutic, ie. post-exposure prophylactic.

**Supplementary Figure 4.**

Supplementary Figure 4. **Kinetics of lung pathology and change in relative lung weight of SARS-CoV-2 infected hamsters.**

(**A**) Body weight loss on D49 in the control groups (separated unvaccinated (C) and adjuvant-only (A) controls) and in the prophylactic and therapeutic settings.

(**B**) Number of animals with severe (all symptoms serious, black) or mild (some lung parameters with lower score, grey) disease as scored and detailed in Suppl. Fig. 5. P+T groups were grouped in order to illustrate the strong difference between vaccinated (P+T) and Control (C) animals in disease severity.

Lung pathology was evaluated as relative lung weight (**C**), lung score (**D**), and lung lesion (**E**) on D44, D46 and D49. (**C**) Relative lung weight illustrated as percentages of body weight ((lung weight/body weight)*100) from D44 to D49. (**D**) The left lobe of the lung was scored for the presence and severity of symptoms (see Materials and Methods). Data are presented as sum of the score of these disease parameters (lung score). (**E**) The whole lung tissue sample (all lobes) was evaluated via visual observation by an independent pathologist and results provided as the % of lung area affected (lung lesion).

Groups were compared by one-tailed Mann-Whitney test (pairwise comparison) and considered statistically significant in case of p<0.05.

**Supplementary Figure 5.**

Supplementary Figure 5. **Summary of lung histopathology analysis (lung score).** Lungs were examined and scored for the presence or absence of alveolar edema, alveolar hemorrhage, and type II pneumocyte hyperplasia (0 = no, 1 = yes). The degree and severity of inflammatory cell infiltration and damage in alveoli, bronchi/bronchioles were scored for alveolitis and bronchitis/bronchiolitis: 0 = no inflammatory cells, 1 =few inflammatory cells, 2 = moderate number of inflammatory cells, 3 = many inflammatory cells. Extent of peribronchial/perivascular cuffing: 0 = none, 1 = 1–2 cells thick, 2 = 3–10 cells thick, 3 = over 10 cells thick. Additionally, the extent of alveolitis/alveolar damage was scored per slide: 0 = 0%, 1 =<25%, 2 =25–50%, 3 =>50%. The cumulative score (sum) for the above parameters provided the total lung score, with a possible maximum score of 18.

Symptoms used to evaluate lung damage are illustrated per animal with colour code: BLUE: score 0/1 or no symptoms (NO, in case of alveolar edema, alveolar hemorrhage, hyperplasia), RED: score 2/3 or presence of symptoms (YES, in case of alveolar edema, alveolar hemorrhage, hyperplasia).

**SUPPLEMENTARY TABLE**

Supplementary Table 1. PolyPEPI-SCoV-2 peptides (30-mers) and the comprised 9-mer CD8+ T cell epitopes (bold) used for immunogenicity testing.

| SARS-CoV-2 fragment (aa position) | ID | Peptide 30-mer, embedded 9-mer is bold |
| --- | --- | --- |
| S (35-64) | S2 | GVYYPDKVFRSSVLH**STQDLFLPF**FSNVTW |
| S (253-282) | S5 | DSSSGWTAGAAAYYVG**YLQPRTFLL**KYNEN |
| S (893-922) | S9 | ALQIP**FAMQMAYRF**NGIGVTQNVLYENQKL |
| N (36-65) | N1 | RSKQRRPQGLPN**NTASWFTAL**TQHGKEDLK |
| N (255-284) | N2 | SKKPRQKRTAT**KAYNVTQAF**GRRGPEQTQG |
| N (290-319) | N3 | ELIRQGTDYKHWPQIAQ**FAPSASAFF**GMSR |
| N (384-413) | N4 | QRQKKQQTVT*LLPAAD*LDD**FSKQLQQSM**SS |
| M (93-122) | M1 | LSYFIASF**RLFARTRSM**WSFNPETNILLNV |
| E (45-74) | E1 | NIVNVSLVKPSF**YVYSRVKNL**NSSRVPDLL |
